# The effect of arteriovenous fistula longevity preservation exercise program combined with video-assisted patient education on fistula maturation for maintenance hemodialysis patients

**DOI:** 10.3389/fmed.2026.1788809

**Published:** 2026-05-20

**Authors:** Shimiao Luo, Xusheng Liu, Qizhan Lin, Zhicong Zhong, Yuchi Wu, Yuling Ye, Junyu Huang, Jingxia Lin, Meizhen Lin

**Affiliations:** 1 Hunan University of Chinese Medicine, Department of Hemodialysis, The Second Affiliated Hospital of Guangzhou University of Chinese Medicine, Dade Road General Hospital, Guangdong Provincial Hospital of Chinese Medicine, Guangzhou, China; 2 Department of Hemodialysis, The Second Affiliated Hospital of Guangzhou University of Chinese Medicine, Dade Road General Hospital, Guangdong Provincial Hospital of Chinese Medicine, Guangzhou, China; 3 Department of Hemodialysis, The Second Affiliated Hospital of Guangzhou University of Chinese Medicine, Fangcun Branch of Guangdong Provincial Hospital of Chinese Medicine, Guangzhou, China; 4 Department of Hemodialysis, The Second Affiliated Hospital of Guangzhou University of Chinese Medicine, University Town Branch of Guangdong Provincial Hospital of Chinese Medicine, Guangzhou, China

**Keywords:** arteriovenous fistula, exercise training, fistula maturation, hemodialysis, video-assisted education

## Abstract

**Objectives:**

This study aimed to investigate the efficacy of arteriovenous fistula (AVF) exercise training combined with video-assisted patient education on AVF maturation in adults undergoing maintenance hemodialysis (MHD).

**Methods:**

A prospective non-randomized controlled trial was conducted at Guangdong Provincial Hospital of Chinese Medicine from September 2023 to November 2024. A total of 88 eligible patients with newly created forearm AVFs were recruited and divided into an intervention group (*n* = 43) and a control group (*n* = 45) according to their willingness to perform novel or traditional exercise training. The control group received standard postoperative care, while the intervention group underwent a structured 8-week program that combined supervised AVF exercise training guided by Chinese Medicine theory with video-assisted feedback education. Primary outcomes included AVF maturation rate, time to maturation, and ultrasonographic parameters (brachial artery blood flow, arterial/venous inner diameter, AVF depth). Secondary outcomes included AVF-related complication rates.

**Results:**

At 8 weeks, the intervention group demonstrated significantly higher brachial artery flow (*P* < 0.001), larger brachial artery inner diameter (*P* < 0.001), and venous diameter (*P* < 0.001), alongside reduced fistula depth (*P* < 0.001). The intervention group also demonstrated higher maturation rates (*P* = 0.030) and shorter median maturation time (*P* = 0.040) compared to the control group. No significant differences in complication rates were observed.

**Conclusion:**

An optimized exercise training program, combined with video-assisted education, enhances fistula maturation parameters and accelerates functional readiness for hemodialysis, suggesting its potential as a complementary strategy to standard AVF care in MHD populations.

**Clinical trial registration:**

https://www.chictr.org.cn/index.html, identifier ChiCTR2400080010.

## 1 Introduction

Maintenance hemodialysis (MHD) remains a cornerstone therapy for end-stage renal disease (ESRD), with reliable vascular access serving as a critical determinant of treatment efficacy (1). The autologous arteriovenous fistula (AVF), recognized as the first-line long-term vascular access by the Kidney Disease Outcomes Quality Initiative (KDOQI), offers superior longevity, lower infection risks, and reduced healthcare costs compared to synthetic grafts or central venous catheters (2, 3). Studies have further confirmed the clinical advantages of AVF and emphasized that appropriate vascular access selection and care are crucial for optimizing patient outcomes (4, 5). Beyond its mechanical advantages, emerging evidence suggests that AVF placement mitigates oxidative stress and chronic inflammation in MHD patients, thereby potentially attenuating dialysis-related complications (6–8).

Despite these benefits, 28–53% of AVFs fail to achieve functional maturation (9, 10). Immature AVFs require the use of temporary catheters, elevating risks of central venous stenosis, bloodstream infections, and mortality (2, 11–13). This underscores the need for interventions to enhance early AVF maturation and optimize dialysis readiness.

Current guidelines emphasize postoperative exercise regimens to accelerate AVF maturation by enhancing arterial blood flow and reducing thrombosis risks (14, 15). However, conventional exercise protocols often prove impractical for MHD patients, who typically exhibit reduced physical capacity and cardiovascular reserve (16, 17). Low-to-moderate intensity exercises, particularly those targeting upper limb mobility without excessive hemodynamic stress, have emerged as viable alternatives for this population (18).

Baduanjin is a traditional mind–body exercise rooted in traditional Chinese medicine (TCM), widely practiced in communities across China and increasingly adopted in other countries. With a history spanning over 800 years, this traditional practice is based on Qigong principles and promotes harmony between the body and mind through slow, rhythmic movements (incorporating aerobic exercise, flexibility training, and isometric resistance exercises), deep breathing techniques, and mental regulation (19). The practice consists of eight simple and easy-to-learn movements that integrate breath control, gentle and graceful body movements, and a relaxed mental state. Traditionally, it is believed to regulate yin and yang, cultivate vital energy (*qi*), unblock meridians, and promote the circulation of *qi* and blood (20, 21). Recent studies have demonstrated Baduanjin’s efficacy in improving fatigue, psychological distress, and sleep quality in MHD patients, while also enhancing exercise tolerance—a critical factor in AVF maintenance (22–25). Its biomechanical characteristics, including rhythmic stretching and rotational movements, may increase endothelial shear stress and improve vascular compliance without compromising fistula integrity (26–28).

Although Baduanjin has been shown to have rehabilitative benefits in MHD patients, it was not specifically designed for AVF conditioning. Therefore, based on the theoretical framework and movement principles of Baduanjin, and incorporating the vascular access characteristics of MHD patients, our research team independently developed a specialized exercise protocol termed the “AVF Longevity Preservation Exercise Program.” This protocol retains the core advantages of Baduanjin, including low intensity and integrated body–mind regulation, while allowing flexible execution in either a standing or sitting position based on the patient’s physical condition. It provides targeted optimization of upper-limb hemodynamic stimulation and aims to translate the systemic benefits of traditional rehabilitative exercise into direct improvements in AVF maintenance.

Complementing such exercise interventions, video-assisted patient education has gained traction as a scalable strategy to improve rehabilitation adherence and technique mastery. Real-time visual feedback enables precise correction of exercise postures, potentially amplifying therapeutic effects while reducing injury risks (29, 30).

Building upon these insights, this study innovatively integrates adapted Baduanjin Exercises with structured video-assisted exercise guidance to create a tailored AVF rehabilitation protocol. Through comparative clinical evaluation, we aim to determine whether this multi-modal intervention enhances AVF maturation metrics compared to standard care, thereby addressing a critical gap in postoperative vascular access management.

## 2 Methods

### 2.1 Study design and ethical compliance

This prospective non-randomized controlled trial was conducted within the Department of Nephrology at Guangdong Provincial Hospital of Chinese Medicine. The protocol underwent rigorous ethical review and received formal approval from the Ethics Committee of Guangdong Provincial Hospital of Chinese Medicine (No. BF2022-259-01). In compliance with international standards for clinical research, the study was prospectively registered with the Chinese Clinical Trial Registry (ChiCTR2400080010) and strictly adhered to the ethical principles of the Declaration of Helsinki.

### 2.2 Participant recruitment

This study enrolled adults (≥18 years) with ESRD who received newly created arteriovenous fistulas (AVFs) for hemodialysis access. All AVFs were created on the non-dominant arm. The mean preoperative vein diameter was 2.82 mm and artery diameter was 1.85 mm. The radial-artery-to-cephalic-vein end-to-side anastomosis was used.

Inclusion criteria: (1) Normal neurological status and muscle strength in the fistula arm; (2) Being able to follow prescribed postoperative exercise regimens by assessment; (3) Sufficient cognitive function to provide written informed consent.

Exclusion criteria: Patients were excluded if they had medical conditions preventing safe participation in AVF rehabilitation, including: (1) Active bleeding from surgical sites; (2) Other postoperative complications restricting physical therapy.

Patients with diabetes or other comorbidities were not excluded, as the aim was to reflect the real-world hemodialysis population.

### 2.3 Termination of protocol

Study participation was discontinued under the following circumstances: (1) Fistula dysfunction or thrombosis; (2) Critical data unavailable due to protocol deviations; (3) Participant attrition (e.g., due to kidney transplantation); (4) Participant-initiated withdrawal of consent.

### 2.4 Allocation and interventions

Eligible participants were introduced to both conventional AVF exercise and the Optimized Exercise Training Program. They were divided into two groups according to their own choice. Patients who chose to participate the Optimized Exercise Training Program were assigned to the intervention group, while those who opted for the conventional exercise plan were placed in the control group. Both groups were provided with routine care and sufficient dialysis treatment.

The routine care for AVFs typically included preoperative guidance, psychological support, postoperative wound inspection, and monitoring and management of surgical bleeding. For all patients in both the intervention and control groups, functional exercises of the operated limb were initiated 1 week postoperatively if no evidence of bleeding or infection was observed. Prior to discharge, patients received detailed instructions which included guidance on identifying potential complications during the maturation phase of the AVF, appropriate measures to address these issues, and scheduling of follow-up appointments. During the study, no bleeding or infection was observed at this time point in any participant. Therefore, the timing of exercise initiation was identical across all patients in both groups, and no deviations occurred due to clinical considerations.

#### 2.4.1 Conventional AVF exercise

Twenty four hours after surgery, patients were instructed to perform fingertip movements, such as simulating playing the piano. 3–7 days after surgery, patients performed wrist movements, such as bending and extending the wrist. After that, patients performed grip exercises, such as fist gripping or squeezing a rubber ball, for 1–2 min per session, repeated 10–20 times daily.

#### 2.4.2 Optimized exercise training program

**FIGURE 1:**
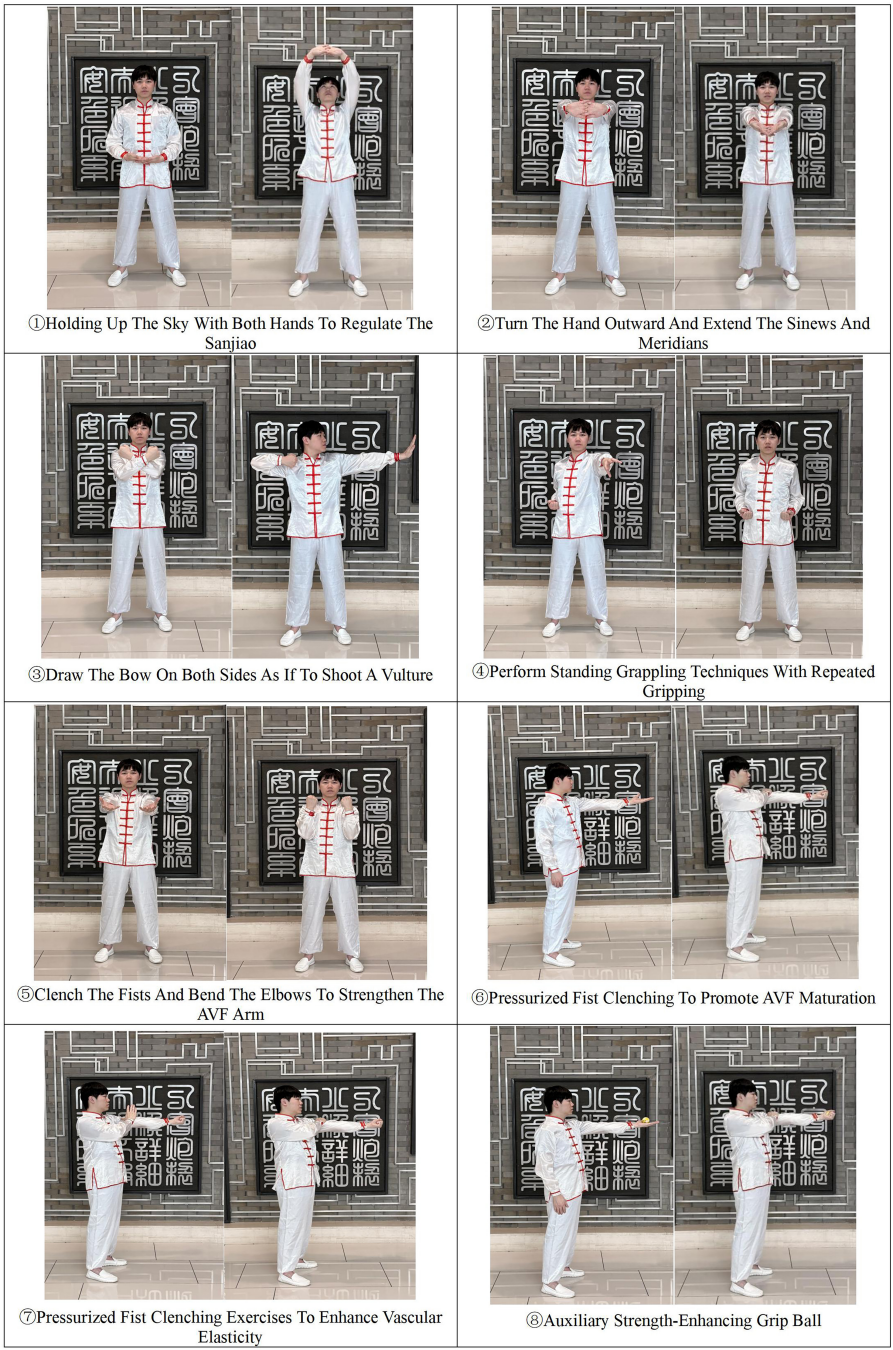
Examples of movements of each action.

This intervention consisted of an AVF longevity preservation exercise program, adapted from Baduanjin, combined with video-assisted patient education. The AVF Longevity Preservation Exercise Program has been registered for national copyright protection at the China Copyright Protection Center (Registration No.: Guozuo Dengzi-2025-I-00261346). The detailed protocol of the exercise program is described as follows: at start, patients should stand with feet shoulder-width apart, with arms relaxed at the sides, and regulate breathing through three cycles of deep inhalation and exhalation. Then, perform the following eight motions accordingly: (1) Holding Up The Sky With Both Hands To Regulate The Sanjiao: raise both hands with the arms internally rotated and the palms facing upward; (2) Turn The Hand Outward And Extend The Sinews And Meridians: rotate hands to stretch tendons and veins; (3) Draw The Bow On Both Sides As If To Shoot A Vulture: curl the right hand into a claw-like shape and pull it to the front of the shoulder. Simultaneously, push the left palm outward to the left side. Alternate this movement between the left and right sides; (4) Perform Standing Grappling Techniques With Repeated Gripping: perform standing captures and repetitive gripping; (5) Clench The Fists And Bend The Elbows To Strengthen The AVF Arm: clench fists and bend elbows to strengthen the fistula arm; (6) Pressurized Fist Clenching To Promote AVF Maturation: the healthy hand firmly grips the fistula arm, while the hand of the fistula arm repetitively makes and releases a fist three times; (7) Pressurized Fist Clenching Exercises To Enhance Vascular Elasticity: The healthy hand firmly grips the fistula arm, and the first of the fistula arm is clenched forcefully for 3–5 s before releasing; (8) Auxiliary Strength-Enhancing Grip Ball: grip a ball with the AVF arm, hold for 3–5 s. Finally, return arms to their original position and regulate breathing through three cycles of inhalation and exhalation. The exercise program was designed to optimize vascular access maturation and functional improvement. Patients were instructed to perform the AVF exercises three times daily, completing three sets of each movement, over an 8-week intervention period. As shown in Figure 1, a pictorial illustration depicting examples of each action was created.

The video-assisted exercise guidance program for AVFs was structured as follows:

(1) Preoperative preparation phase: Patients scheduled for AVF surgery were introduced to the importance of AVF exercises. Educational videos were played on ward televisions to provide patients with a basic understanding of the exercise process and key techniques.

(2) Postoperative guidance phase: Starting on the third day after surgery, nursing staff began guidance for AVF exercises. First, patients performed AVF exercises under video guidance and the nursing staff observed to ensure proper and accurate execution of movements. Subsequently, nursing staff corrected and guided patients on any inaccuracies. Finally, focusing on patients’ errors or deviations, nursing staff provided detailed instruction and demonstration until patients demonstrated full mastery of all exercise techniques.

(3) Daily practice phase: The instructional video for AVF exercises was cyclically broadcast on ward televisions each day. This enabled patients to access and review the content at their convenience, facilitating continuous learning. Patients were encouraged to engage in independent practice of AVF exercises, at a minimum frequency of three sessions per day. Nursing staff conducted routine ward rounds to observe patients’ exercise performance, correct errors promptly, and ensure adherence to proper exercise techniques and optimal effectiveness.

(4) Post-discharge home training: After discharge, patients were provided with a video demonstrating AVF exercises to facilitate continued practice at home. Patients were advised to keep an exercise log recording the timing of exercise and condition of the AVF for each session. Additionally, patients were instructed to perform self-assessment of the AVF at home three times daily.

(5) Home supervision and follow-up: Family members were assigned to supervise patients’ exercise implementation at home. Responsible nurses conducted regular follow-ups via telephone, home visits, or online platforms to review patients’ exercise logs, assess their mastery and practice adherence, and to provide necessary guidance and support. Patients were encouraged to promptly report any problems or discomfort encountered during exercise to responsible nurses, seeking for timely adjustments to the exercise program.

(6) Psychological support: Throughout the rehabilitation process, patients received comprehensive psychological support and encouragement.

### 2.5 Outcomes

#### 2.5.1 Ultrasound measurements at 8 weeks

At 8 weeks postoperatively, ultrasonography was performed in both groups using a Sonosite M-Turbo ultrasound system (SonoSite, Inc., Bothell, WA, United States) equipped with a linear array transducer (HFL38x, 6–13 MHz). The following parameters were measured: brachial artery flow, brachial artery inner diameter (Brachial ID), anastomotic inner diameter (Anastomotic ID), AVF inner diameter (AVF ID, namely the inner diameter of the cephalic vein in the forearm), and AVF depth (the distance from the target cannulation point of vein to the skin). All measurements were performed by a certified sonographer who was blinded to group allocation. Patients were instructed not to disclose their group assignment to the sonographer before or during the examination. The sonographer was not involved in the intervention or outcome assessment process. Each parameter was measured three times, and the average value was used for analysis. All images were stored, and a second blinded investigator independently reviewed the measurements to ensure reliability.

#### 2.5.2 Time to maturity and dialysis blood flow

The cannulation of AVF was initiated when it met the following criteria: brachial artery flow exceeding 500 mL/min, AVF inner diameter > 5 mm, and AVF depth from the skin surface < 6 mm (30). The clinical maturity of AVFs was defined as the ability of the AVF to support effective hemodialysis with two needles for consecutive six dialysis sessions. The time from AVF creation to clinical maturity were calculated. The blood flow rate of the first dialysis and that at 4, 8, and 12 weeks post-dialysis initiation were also recorded.

#### 2.5.3 Complications

Any adverse events in the maturation process were recorded and the incidences of complications related to AVF exercise were compared between the two groups. Potential complications included AVF early dysfunction, subcutaneous hematoma, pseudoaneurysm, bleeding, and thrombosis.

### 2.6 Statistical analysis

Statistical analysis was performed using SPSS 28.0. Continuous variables were expressed as mean ± standard deviation (SD). For comparisons between the two groups, independent-sample *t*-tests were used, with Levene’s test for equality of variances applied to determine whether the variances were homogeneous. If variances were unequal, a corrected *t*-test was employed. Cohen’s *d* was calculated to estimate the effect size of differences, with Cohen’s *d* = 0.2 considered a small effect, d = 0.5 a medium effect, and d ≥ 0.8 a large effect. *Post-hoc* power analyses were performed using G*Power version 3.1 with α = 0.05 (two-tailed). Categorical variables were presented as frequencies and percentages [*n* (%)]. Comparisons between groups were conducted using chi-squared tests or Fisher’s exact test. A two-tailed *p*-value of < 0.05 was considered statistically significant for all analyses.

### 2.7 Sample size

In this study, the sample size was estimated using a method for comparing rates between two independent samples, with the AVF maturation rate as the primary outcome indicator. With reference to previous literature (31), it was assumed that the AVF maturation rate in the control group was 50%, and the expected maturation rate in the intervention group was 80%. A two-tailed test was set with a significance level (α) of 0.05 and a power of the test (1-β) of 80% (β = 0.2).

According to the formula for sample size estimation:


$$n=\frac{{\left(z_{1-a/2}+Z_{1-\beta }\right)}^{2}\cdot \left(p_{c}\,\left(1-p_{c}\right)+p_{t}\,\left(1-p_{t}\right)\right)}{{\left(p_{t}-p_{c}\right)}^{2}}$$

The calculation showed that approximately 36 samples were required for each group. Considering a potential dropout rate of 10%, the minimum sample size for each group was finally set at 40 cases, totaling 80 cases. The final sample size of this study was 88 cases, including 45 cases in the control group and 43 cases in the intervention group.

## 3 Results

**FIGURE 2:**
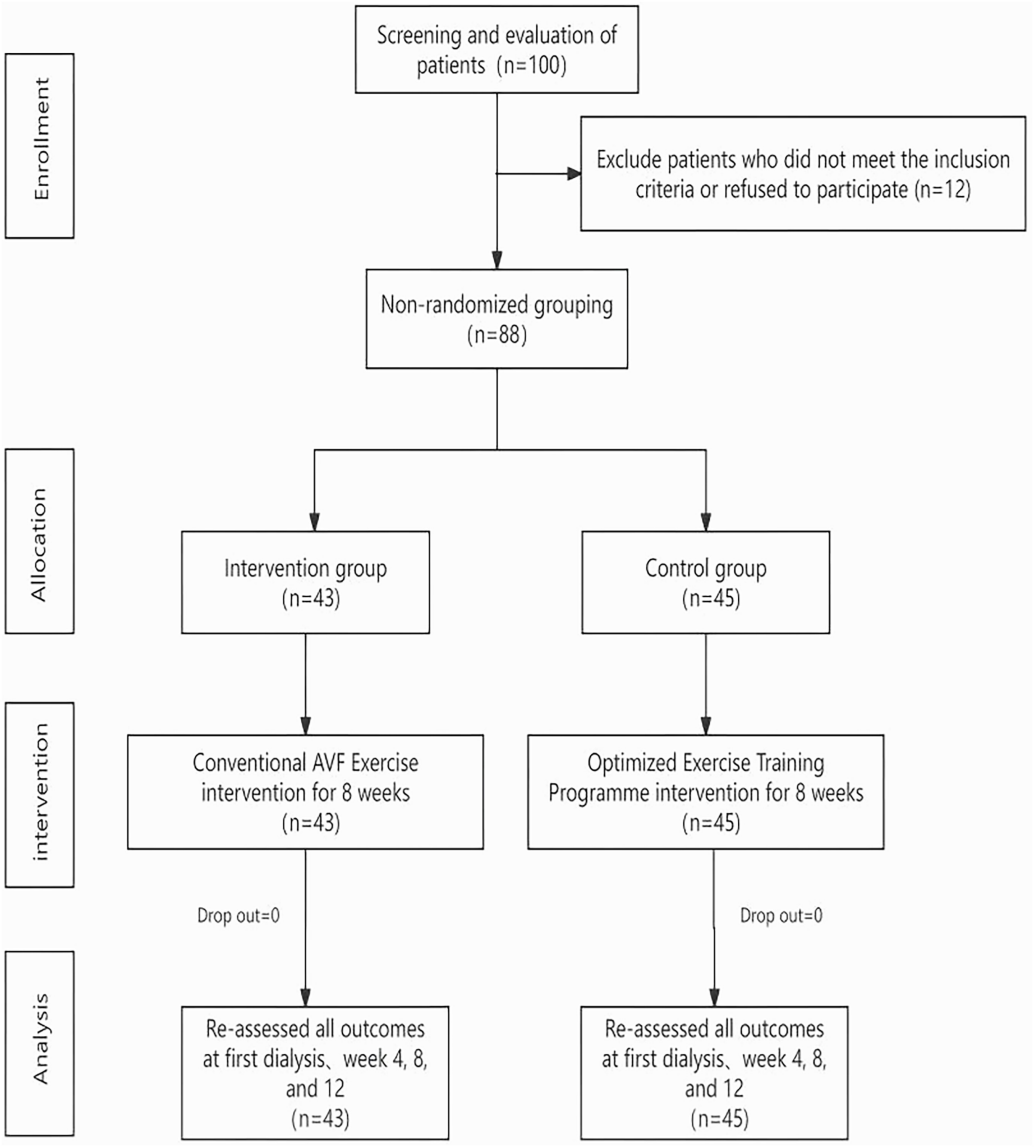
Consolidated standards of reporting trials (CONSORT) diagram of study procedure.

From September 2023 to November 2024, 100 patients were assessed for eligibility. After excluding 12 patients who did not meet inclusion criteria or declined participation, 88 were ultimately enrolled in the study. Among them, 43 were assigned to the intervention group while 45 to the control group. No cases of dropout or withdrawal were observed in either group by the end of the study. The study procedure was illustrated by the Consolidated Standards of Reporting Trials (CONSORT) flow diagram (32) (Figure 2). The demographic characteristics of the two groups was comparable, without statistically significant difference (*P* > 0.05). Detailed information is provided in Table 1.

**TABLE 1:** Participants characteristics at baseline (*N* = 88).

Variables	Intervention group (*n* = 43)	Control group (*n* = 45)	*t*/χ^2^	*P*
Gender			0.001	0.973
Male	25	26		
Female	18	19		
Age (years)	62.05 ± 14.35	58.27 ± 14.17	1.243	0.217
Marital status			3.137	0.208
Single	6	6		
Married	32	38		
Divorced/Widowed	5	1		
Education level			2.556	0.464
Primary school and below	13	10		
Middle school	8	14		
High school or secondary school	14	11		
College or above	8	10		
Underlying disease for CKD			0.466	0.926
Chronic nephritis	5	7		
Diabetic nephropathy	18	19		
Hypertensive nephropathy	13	12		
Others	7	6		

### 3.1 Comparison of ultrasonographic findings of AVF between groups

At 8 weeks post-surgery, the intervention group showed significantly higher brachial artery blood flow (*P* < 0.001, *d* = 0.924), brachial artery inner diameter (*P* < 0.001, *d* = 1.465), and AVF inner diameter (*P* < 0.001, *d* = 1.059), as well as significantly smaller AVF depth (*P* < 0.001, *d* = −0.795), compared to the control group. No significant difference was found in anastomotic inner diameter (*P* = 0.316, *d* = 0.214). Statistical power exceeded 0.990 for all significant outcomes (see Table 2).

**TABLE 2:** Comparison of ultrasonographic findings of AVF between the two groups.

Variables	Intervention group (*n* = 43)	Control group (*n* = 45)	*t*	*P*	Cohen’s d	Power
Brachial artery blood flow (mean ± SD, mL/min)	828.4 ± 325.85	602 ± 126.634	4.332	< 0.001	0.924	> 0.990
Brachial ID (mean ± SD, mm)	5.34 ± 0.89	4.08 ± 0.83	6.881	< 0.001	1.465	> 0.990
Anastomotic ID (mean ± SD, mm)	3.31 ± 0.74	3.16 ± 0.66	1.009	0.316	0.214	0.120
AVF ID (mean ± SD, mm)	5.29 ± 1.00	4.27 ± 0.93	4.865	< 0.001	1.059	> 0.990
Depth of AVF (mean ± SD, mm)	2.62 ± 1.11	3.67 ± 1.49	−3.716	< 0.001	−0.795	> 0.990

ID, inner diameter; AVF, arteriovenous fistula; mean ± SD, mean ± standard deviation. *by independent-sample t-tests.

### 3.2 Comparison of AVF maturation rate and time to maturity between groups

The maturation rate of AVF was significantly higher in the intervention group compared to the control group (59.18% vs. 40.82%, χ^2^ = 4.713, *P* = 0.030). Additionally, the time to AVF maturation was significantly shorter in the intervention group than that in the control group (78.26 ± 34.52 days vs. 91.44 ± 24.15 days, *t* = −2.084, *P* = 0.040). These results are summarized in Table 3.

**TABLE 3:** Comparison of AVF maturation rate and maturation time between groups.

Groups	AVF maturation rate [n(%)]	AVF maturation time (days, mean ± SD)
Intervention group	29(59.18)	78.26 ± 34.52
Control group	20(40.82)	91.44 ± 24.15
t/χ^2^	4.713	−2.084
*P*	0.030	0.040

Mean ± SD, mean ± standard deviation.

### 3.3 Comparison of dialysis blood flow between groups

**FIGURE 3:**
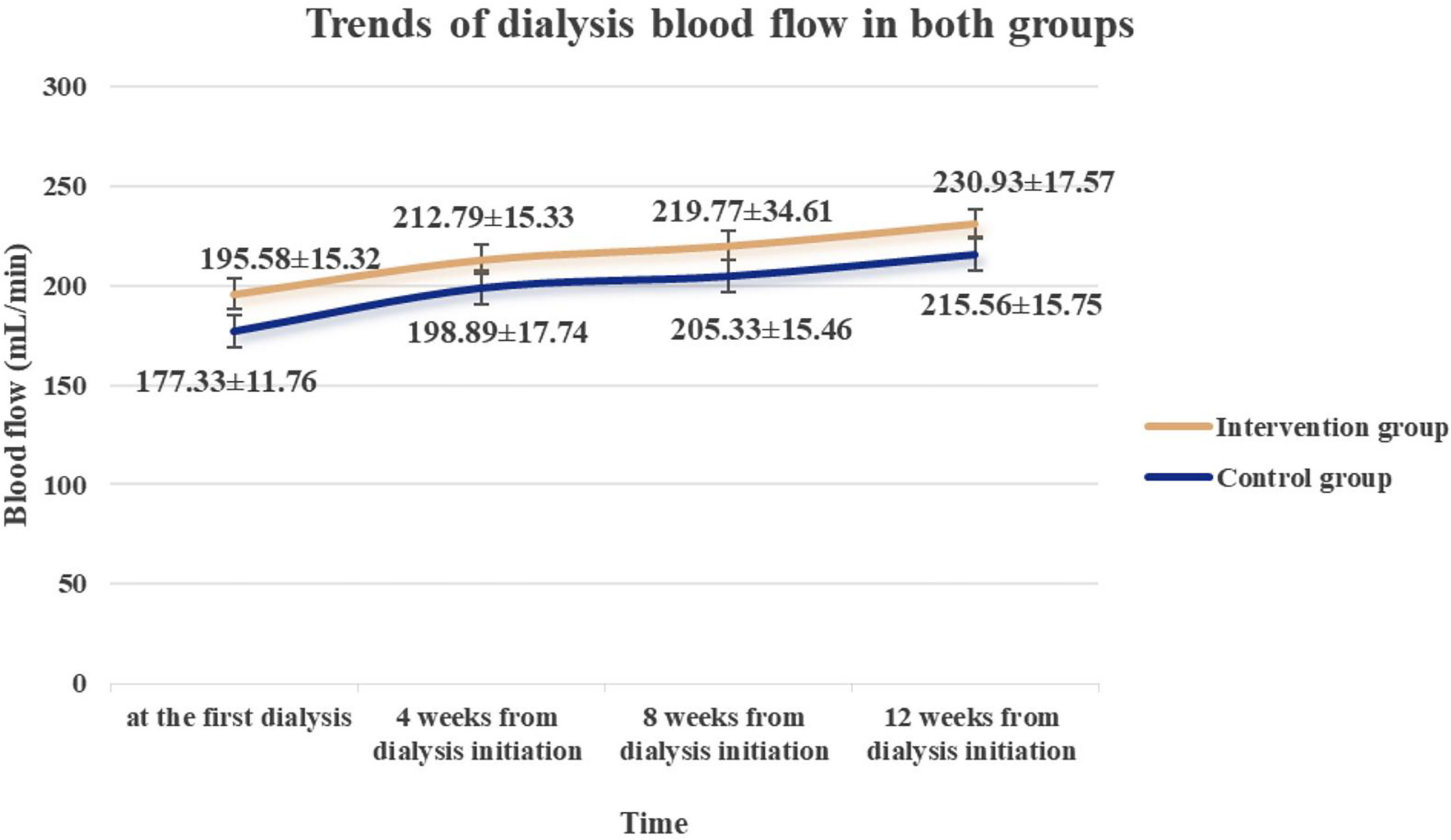
Trends of dialysis blood flow over time in the intervention and control groups.

The blood flow on dialysis of the intervention group was significantly higher than that of the control group at the first dialysis session, as well as at 4, 8, and 12 weeks post-dialysis initiation. These differences were statistically significant across all time points (*P* < 0.01). A detailed comparison of dialysis blood flow between the groups is provided in Figure 3. Table 4 shows the trends of dialysis blood flow in both groups of patients.

**TABLE 4:** Comparison of dialysis blood flow between groups at different time.

Blood flow (mean ± SD)	Intervention group (*n* = 43)	Control group (*n* = 45)	*t*	*P*	Cohen’s *d*	Power
At the first dialysis(mL/min)	195.58 ± 15.32	177.33 ± 11.76	6.285	< 0.001	1.340	> 0.990
4 Weeks from dialysis initiation (mL/min)	212.79 ± 15.33	198.89 ± 17.74	3.926	< 0.001	0.837	> 0.990
8 Weeks from dialysis initiation (mL/min)	219.77 ± 34.61	205.33 ± 15.46	2.545	0.013	0.543	0.72
12 Weeks from dialysis initiation (mL/min)	230.93 ± 17.57	215.56 ± 15.75	4.327	< 0.001	0.923	> 0.990

### 3.4 Incidence of complications between groups

The incidence of AVF complications was lower in the intervention group (4.63%, 2/43) compared to the control group (17.56%, 7/45). However, the difference in complication rates between the two groups did not reach statistical significance (χ^2^ = 2.848, *P* = 0.092).

## 4 Discussion

The establishment of a functional AVF remains the gold standard for vascular access in hemodialysis patients. However, its clinical success depends heavily on achieving adequate vascular remodeling and maintaining long-term patency. Our findings demonstrate that an 8-week structured exercise protocol combined with video-assisted education significantly improved AVF maturation parameters compared to standard care, providing evidence that targeted interventions can optimize access outcomes in vulnerable populations. Several factors may explain these findings. First, functional exercise itself has been shown to promote early AVF maturation. However, in clinical practice, the lack of standardized protocols and poor patient adherence often prevent its physiological benefits from being fully realized, leading to delayed fistula maturation and an increased incidence of complications (33–35). The 8-week structured exercise program employed in this study, with clearly defined frequency, intensity, and procedures, enabled the full physiological benefits of functional exercise to be achieved. Second, scientific and precise nursing interventions have been proven to effectively promote fistula maturation and extend its lifespan, with improving patients’ self-care ability being central to achieving this goal (36). However, existing studies indicate that self-care ability regarding AVF among maintenance hemodialysis patients is generally at a low-to-moderate level (37). Dilbilir et al. demonstrated that structured AVF nursing training significantly improves patients’ self-care behaviors (38). In this study, the introduction of video-assisted educational guidance, through intuitive and clear operational demonstrations, helped patients correctly master the exercise methods, thereby improving intervention adherence and execution quality. This provided a solid foundation for the successful implementation of the structured exercise program.

This study further observed clear hemodynamic benefits. The observed hemodynamic improvements in the intervention group—particularly the 37.6% greater brachial artery flow and 30.9% larger venous diameter—align with recent murine models showing that mechanical forces from repetitive exercise may enhance venous outward remodeling through endothelial shear stress modulation (39). Notably, the intervention group achieved a reduction in median time to maturation of 13 days (14.4% acceleration), suggesting that early biomechanical stimulation could counteract venous contractile phenotype shifts observed in delayed maturation cases. These findings extend previous clinical reports by demonstrating that Chinese Medicine-inspired exercises—when systematically applied with compliance monitoring—can achieve hemodynamic benefits comparable to pharmacological interventions, without increasing complication risks.

The biomechanical efficacy of our eight-step exercise protocol likely stems from its phased approach combining systemic preparation with localized vascular stimulation. Initial maneuvers targeting *qi* regulation and myofascial relaxation (Steps 1–4) may enhance regional perfusion through autonomic modulation, as evidenced by recent acupuncture research (40). Subsequent pressure-application exercises (Steps 5–8) generate precisely timed venous distension cycles (peak pressures 40–60 mmHg), a mechanical stimulus shown to upregulate endothelial nitric oxide synthase and MMP-2 activity, both critical for venous wall remodeling (41, 42). This mechanistic synergy between rhythmic compression and blood flow acceleration appears to promote optimal neointimal hyperplasia—sufficient for venous dilatation yet avoiding excessive stenosis (43). Contrary to traditional resistance training paradigms (44), our Chinese medicine-inspired protocol emphasizes dynamic pressure modulation over static strength building. This approach may reduce turbulent flow patterns implicated in access thrombosis (45).

According to recent surveys, only 30% of patients consistently adhere to prescribed exercise regimens in clinical practice (30), representing a critical barrier in vascular access care. The video-assisted guidance education component was designed to address this barrier (46). Our multimedia education approach may have enhanced procedural fidelity and patient engagement—key determinants of exercise intervention success. This synergy between biomechanical and behavioral interventions creates a comprehensive strategy addressing both biological and psychosocial dimensions of AVF maturation.

## 5 Limitations

Several limitations exist in this study. First, our single-center design and 8-week endpoint preclude assessment of long-term patency outcomes. Second, while Doppler ultrasound provides objective hemodynamic data, the lack of histopathological correlation limits mechanistic confirmation. Third, the non-randomized allocation scheme may introduce unmeasured confounders that interfere with the results and cause selection bias. Although baseline characteristics were comparable between groups, unmeasured residual confounding may still exist and could influence the findings. Fourth, this study included only patients who were able to complete the full arteriovenous fistula exercise protocol, excluding those unable to perform the exercises due to limited limb mobility or severe frailty. Therefore, the applicability of this intervention in such populations remains unclear. Future studies should control for more confounding factors and incorporate multicenter randomized controlled trials with advanced imaging and molecular biomarkers to further elucidate the exercise-remodeling relationship. In addition, for patients who are unable to perform the full arteriovenous fistula exercise regimen, future research should develop and validate modified or simplified versions of the protocol to broaden its applicability to a wider patient population. Additionally, as a traditional Chinese method, caution is needed when extrapolating our findings to other cultural backgrounds, and cross-cultural validation is warranted.

## 6 Conclusion

Structured exercise combined with multimedia education accelerates AVF maturation through hemodynamic optimization, offering a safe adjunct to current postoperative protocols. This exercise program is safe, low-intensity, easy to implement, and requires no expensive equipment. Therefore, it is recommended for clinical promotion as a complementary intervention to promote AVF maturation. However, for patients with limited limb mobility or severe frailty who are unable to perform the full exercise protocol, individualized gentle or simplified versions should be explored in clinical practice, and their effects warrant further investigation.

Additionally, this study has limitations including its single-center design, short-term follow-up, and non-randomized allocation. Future studies are needed to further validate the long-term effects and generalizability of this intervention.

## Data Availability

The raw data supporting the conclusions of this article will be made available by the authors, without undue reservation.
